# A Department of Public Health

**Published:** 1897-12

**Authors:** 


### Abstracts and Selections.

---

### A Department of Public Health.

The *Journal* of the *American Medical Association* (Nov. 20, '97), says that the yellow fever outbreak in the South this year has served a good purpose in that it has demonstrated the inefficiency of "the makeshift methods" of "utilizing the expedients of a special bureau created for an entirely different purpose, clinical only, and not sanitary in its aims; the most dreaded of pestilential diseases having actually gained entrance under the very eyes and nose of the very much vaunted guardian"—the Marine Hospital Service. It has demonstrated the necessity of a Department of Public Health, and the American Medical Association, and the American Public Health Association have "united in the opinion and earnest recommendation to Congress that there shall be established a Department of Public Health, the duties of which shall be to collect and diffuse information upon matters affecting the public health, including statistics of sickness and mortality in the several states and territories; the investigation by experimental and other methods of the causes and means of prevention of diseases; the collection of information with regard to the prevalence of infections, contagious and epidemic diseases both in this and other countries; also, the causative and curative influences of climate upon the same; the publication of the information thus obtained in a weekly bulletin; the preparation and promulgation of rules and regulations for securing the best sanitary condition of vessels from foreign ports and for prevention of the introduction of infectious diseases into the United States and their spread from one state to another; that this Department of Public Health shall be under the control and management of a Commissioner of Public Health, who shall be appointed by the president, by and with the consent of the Senate, who shall hold his office for six years, shall be a regularly educated physician of at least ten years' experience, learned in the practice of medicine, in sanitary science, and member of one or more reputable sanitary or medical associations in the United States; and that the Commissioner of Public Health shall semi-annually and at such other time, as he may designate, call to meet in the city of Washington, D. C., an *advisory council* to be composed of the secretary or executive officer of each State and Territorial Board of Health, and an officer learned in the law, detailed by the Attorney-General of the United States from the Department of Justice. A Department of Public Health, controlled and managed by a Commissioner of Public Health, with an advisory council of executive officers of each State and Territorial Board of Health, are the essential features of the scheme of law, which was reported to and accepted by the American Public Health Association and ordered to be transmitted to the Congress of the United States as its voice and opinion. This action now unites it with the American Medical Association. The elaboration of the scheme may be safely trusted to the national law-makers."

